# On the Adjacent Eccentric Distance Sum Index of Graphs

**DOI:** 10.1371/journal.pone.0129497

**Published:** 2015-06-19

**Authors:** Hui Qu, Shujuan Cao

**Affiliations:** 1 Department of Mathematics, Shandong Institute of Business and Technology, Yantai, Shandong, China; 2 School of Mathematical Sciences, Nankai University, Tianjin, China; Mathematical Institute, HUNGARY

## Abstract

For a given graph *G*, *ε*(*v*) and *deg*(*v*) denote the eccentricity and the degree of the vertex *v* in *G*, respectively. The *adjacent eccentric distance sum index* of a graph *G* is defined as $\xi^{sv}(G)=\sum_{v\in V(G)}\frac{\varepsilon (v)D(v)}{deg(v)}$, where $D(v)=\sum_{u\in V(G)}d(u,v)$ is the sum of all distances from the vertex *v*. In this paper we derive some bounds for the adjacent eccentric distance sum index in terms of some graph parameters, such as independence number, covering number, vertex connectivity, chromatic number, diameter and some other graph topological indices.

## Introduction

In theoretical chemistry and biology, molecular structure descriptors or topological indices are used for modeling information of molecules, including physico-chemical, toxicologic, pharmacologic, biological and other properties of chemical compounds. A topological index is a numerical value based on a simple graph, which is an invariant. Topological indices have been found to be useful in chemistry, bioinformatics and network science. Generally, these indices are divided into three classes. One class is degree-base indices [51], such as (general) Randic index [46, 52], (general) zeroth order Randic index [34, 35], Zagreb index [26], ABC index [50]. One class is distance-based indices [61], such as Wiener index [40, 57, 58, 60], Balaban index [4, 5, 16], the Wiener polarity index [24, 49], Szeged index [1], Harary index [2], Hosoya index [32, 33]. The third class is spectrum-based indices, such as graph energy [18, 27, 31, 36, 37, 47, 48], incidence energy [9, 10], matching energy [12, 13, 43], Randic energy [8, 19], HOMO-LUMO index [45], Kirchhoff index [44], graph entropies [11, 14, 15, 20, 21, 42, 65]. Recently, some other indices based on eccentricity have been introduced and investigated by mathematicians, chemists and biologists, which contain eccentric connectivity index [28, 41, 55], eccentric distance sum [29, 38, 62], augmented and super augmented eccentric connectivity indices [3, 25], connective eccentricity index [30, 63, 64], adjacent eccentric distance sum index [53, 54] and so on.

Throughout this paper, graphs considered are simple and connected. Let *G* be a simple connected graph with vertex set *V*(*G*). For a vertex *v* ∈ *V*(*G*), *deg*(*v*) denotes the degree of *v*. For vertices *u*, *v* ∈ *V*(*G*), the *distance* *d*(*u*, *v*) is defined as the length of the shortest path between *u* and *v* in *G* and *D*_*G*_(*v*) (or *D*(*v*) for short) denotes the sum of all distances from *v*. The *eccentricity* *ɛ*(*v*) of a vertex *v* is the maximum distance from *v* to any other vertex. The *radius* *r*(*G*) of a graph is the minimum eccentricity of any vertex, while the *diameter* *D*(*G*) of a graph is the maximum eccentricity of any vertex in the graph. Let *K*_*n*_ and *P*_*n*_ be the complete graph and path graph on *n* vertices, respectively. We write *K*_*n*_ − *ke* for the graph obtained from *K*_*n*_ by deleting *k* independent edges.

In chemistry and biology, people want to find some new molecules with desired physicochemical properties, for example, boiling points, molecular volumes, flavor threshold concentration antiviral activity, energy levels, electronic populations, etc. And these properties can be quantitatively represented by some values of a certain index. The notion of a “topological index” appears first in a paper of the Japanese chemist Hosoya [32], who investigated the surprising relation between the physicochemical properties of a molecule and the number of its independent edge subsets (matchings). A topological index is a map from the set of chemical graphs to the set of real numbers. Therefore a topological index is a numeric quantity that is mathematically derived in a direct and unambiguous manner form the structural graph of a molecule. Since isomorphic graphs possess identical values for any given topological index, these indices are referred to as graph invariants. Topological indices usually reflect both molecular size and shape. The advantage of topological indices is that they may be used directly as simple numerical descriptors in a comparison with physical, chemical, or biological parameters of molecules in quantitative structure-property relationships (QSPR) and in quantitative structure-activity relationships (QSAR). Therefore, one of the most interesting problems on topological indices is to study the extremal properties of topological indices and characterize the extremal structures. Another direction is to study the relation between topological indices and some other graph invariant, which would help people to know the indices better and find more internal information.

The quantification of chemical structures is a basic problem in structure-activity relationships (SAR) which is an important tool for developing safer and potent drugs. The procedure for quantification of chemical structures is by translation of chemical structures into characteristic numerical descriptors (topological indices) [6, 7, 39, 56]. This procedure provides a correlation between quantitative biological activity and qualitative chemical structures. Based on this, Sardana and Madan [53] introduced a novel topological descriptor–adjacent eccentric distance sum index, which is defined as

$$\begin{array}{c} \xi^{sv}\left(G\right)=\sum_{v\in V(G)}\frac{\varepsilon (v)D(v)}{deg(v)}. \end{array}$$

To verify that this index has a vast potential for SAR, Sardana and Madan [53, 54] selected different data sets to investigate some properties of adjacent eccentric distance sum index. In [53], to predict anti-HIV activity of 4,5,6,7-tetrahydro-imidazo-[4, 5, 1−*jk*][1, 4] benzodiazepin-2 (1H)-one (TIBO) derivatives, the authors introduced this index which is computable. Moreover, the authors compared property of the adjacent eccentric distance sum index with that of the eccentric connectivity index and derived some excellent correlations between anti-HIV activity and both the topological descriptors. Their results show that the adjacent eccentric distance sum index has a potential for structure-activity/property studies. As we know, Wiener index is one of the most studied topological indices from the view of theory and applications, see for details [22, 23, 59]. In [54], the authors investigated the relationship of Wiener index and adjacent eccentric distance sum index with the antioxidant activity of nitroxides, as well as their hydroxylamine and amine precursors. In their investigation, authors selected 68 analogues whose antioxidant activity was reported. Firstly they computed the values of the Wiener index and the adjacent eccentric distance sum index for each analogues. Subsequently, each analogue was assigned a biological activity which was then compared with the reported antioxidant activity as *H*_2_ *O*_2_ protection at a concentration of 100 *μ*M. They supposed that compounds with an *H*_2_ *O*_2_ protection of 2.5 or more were active and those with *H*_2_ *O*_2_ protection of less than 2.5 or more were inactive. The overall degree of prediction was derived from the ratio of the total number of compounds predicted correctly to that of the total number of all compounds in both active and inactive ranges. Their results show that a total of 58 out of 68 compounds were classified correctly in both the active and inactive ranges with regard to Wiener index, but for adjacent eccentric distance sum index a total of 59 out of 68 compounds were classified correctly. The overall degree of prediction was found to 0.85 in case of Wiener index and 0.87 in case of adjacent eccentric distance sum index. These results encourage us to study the mathematical properties of this novel topological descriptor.

In this paper we derive some bounds for adjacent eccentric distance sum index in terms of some graph parameters, such as independence number, covering number, chromatic number, diameter and some graph topological indices.

## Methods

Since adding edges would increase the degrees of some vertices and not increase *ɛ*(*v*), *D*(*v*) for any vertex *v* ∈ *V*(*G*), we have the following result.

**Lemma 1** *Let G be a non-complete connected graph and e be an edge in* $\bar{G}$ *(the complement of G). Then* *ξ* ^*sv*^(*G*) > *ξ* ^*sv*^(*G*+*e*).

The following result is immediate from Lemma 1.

**Theorem 1** *Let G be a connected graph on n vertices. Then*

$$\begin{array}{c} \xi^{sv}\left(G\right)\geq n, \end{array}$$

*with equality holding if and only if G* ≅ *K*_*n*_.

*If G* ≠ *K*_*n*_, *then*

$$\begin{array}{c} \xi^{sv}\left(G\right)\geq n-2+\frac{4n}{n-2}, \end{array}$$

*with equality holding if and only if G* ≅ *K*_*n*_−*e*.

A graph is *vertex-transitive* if, for any two vertices *u* and *v*, there is an automorphism *f* of *G* such that *f*(*u*) = *f*(*v*). For a vertex-transitive graph *G*, it is regular and *r*(*G*) = *ɛ*(*w*) for any *w* ∈ *V*(*G*). The *Wiener index* of a graph *G*, denoted by *W*(*G*), is defined as the sum of the distances between all pairs of vertices in graph *G*, that is

$$\begin{array}{c} W\left(G\right)=\sum_{\left\{u,v\}\subseteq V(G\right)}d\left(u,v\right)=\frac{1}{2}\sum_{v\in V(G)}D\left(v\right). \end{array}$$

**Theorem 2** *Let G be a vertex-transitive graph on n vertices with degree δ. Then*

$$\begin{array}{c} \xi^{sv}\left(G\right)=\frac{2r(G)}{\delta }W\left(G\right). \end{array}$$

The *total eccentricity* of the graph *G*, denoted by *ζ*(*G*), is defined as the sum of eccentricities of all vertices of graph *G*, i.e.,

$$\begin{array}{c} \zeta \left(G\right)=\sum_{v\in V(G)}\varepsilon \left(v\right). \end{array}$$

It is sometimes interesting to consider it [17].

**Theorem 3** *Let G be a connected graph on n* ≥ 3 *vertices. Then*

$$\begin{array}{c} \xi^{sv}\left(G\right)\geq \zeta \left(G\right), \end{array}$$

*with equality holding if and only if G* ≅ *K*_*n*_.

*Proof.* By fact that *D*(*v*) ≥ *deg*(*v*) with equality holding if and only if *ɛ*(*v*) = 1 and *deg*(*v*) = *n*−1, i.e., *G* ≅ *K*_*n*_, we have

$$\begin{array}{c} \xi^{sv}\left(G\right)\geq \sum_{v\in V(G)}\frac{\varepsilon (v)deg(v)}{deg(v)}=\sum_{v\in V(G)}\varepsilon \left(v\right)=\zeta \left(G\right). \end{array}$$

An *independent set* of *G* is a subset *S* of *V*(*G*) such that no two vertices in *S* are adjacent in *G*. The *independence number* of *G*, denoted by *α*(*G*), is the size of a maximum independent set of *G*. Let 𝓖(*n*, *α*) be the set of all connected graphs with *n* vertices and independence number *α*. Let *G* ^⋄^ denote the graph $K_{n-\alpha }\vee \bar{K_{\alpha }}$. Let *V*(*G* ^⋄^) = *V*_1_∪*V*_2_, where *V*_1_ = {*v*_1_, *v*_2_, ⋯, *v*_*α*_} is the independent set $\bar{K_{\alpha }}$ of *G* ^⋄^ and *V*_2_ = {*v*_*α*+1_, ⋯, *v*_*n*_} is the vertex set of *K*_*n*−*α*_. Let *e*_1_ be an edge joining two vertices in *V*_1_ and *V*_2_. Let *e*_2_ be an edge incident to two vertices in *V*_2_.

**Theorem 4** *Let G be a connected graph with n vertices and independence number α. Let* $C=\lfloor n-\frac{1+\sqrt{\frac{16n^{2}-47n+34}{n+2}}}{2}\rfloor$ *be an integer. Then*

- $\xi^{sv}(G)\geq n-\alpha +\frac{2\alpha (n+\alpha -2)}{n-\alpha },$ *with equality holding if and only if G* ≅ *G* ^⋄^;
- *if G* ∈ 𝓖_*n*,*α*_∖{*G* ^⋄^}, *then*
  $$\begin{array}{c} \xi^{sv}\left(G\right)\geq \{\begin{array}{cc} \frac{2(n+\alpha -2)(\alpha -1)}{n-\alpha }+\frac{2(n+\alpha -1)}{n-\alpha -1}+\frac{2n}{n-2}+n-\alpha -1 & \;\text{if}\;2\leq \alpha \leq C\text{,}\\ \frac{2\alpha (n+\alpha -2)}{n-\alpha }+\frac{4n}{n-2}+n-\alpha -2 & \;\text{if}\;\alpha \geq C+1\text{,} \end{array} \end{array}$$
  *with equality holding if and only if G* ≅ *G* ^⋄^−*e*_1_ *when* 2 ≤ *α* ≤ *C*; *G* ≅ *G* ^⋄^−*e*_2_ *when* *α* ≥ *C*+1.

*Proof.* Assume that *G*_0_ is a graph with the minimal adjacent eccentric distance sum index among all connected graphs on *n* vertices with independence number *α*. Let *S* be a maximum independent set of *G*_0_. Since adding edges would decrease the adjacent eccentric distance sum index, it follows that any vertex in *V*(*G*_0_)−*S* is adjacent to every vertex in *S* and *G*_0_−*S* is complete. So *G*_0_ ≅ *G* ^⋄^. We have $\xi^{sv}(G^{⋄})=n-\alpha +\frac{2\alpha (n+\alpha -2)}{n-\alpha }$.

By deleting an edge in *G* ^⋄^, we get two graphs up to isomorphism: *G* ^⋄^−*e*_1_, *G* ^⋄^−*e*_2_. Since deleting an edge would increase the adjacent eccentric distance sum index, we only compare *ξ* ^*sv*^(*G* ^⋄^−*e*_1_) with *ξ* ^*sv*^(*G* ^⋄^−*e*_2_).

By some elementary calculations, we have

$$\begin{array}{ccc} \xi^{sv}\left(G^{⋄}-e_{1}\right) & = & \frac{2(n+\alpha -2)(\alpha -1)}{n-\alpha }+\frac{2(n+\alpha -1)}{n-\alpha -1}+\frac{2n}{n-2}+n-\alpha -1\\ \xi^{sv}\left(G^{⋄}-e_{2}\right) & = & \frac{2\alpha (n+\alpha -2)}{n-\alpha }+\frac{4n}{n-2}+n-\alpha -2. \end{array}$$

So we have

$$\begin{array}{c} \xi^{sv}\left(G^{⋄}-e_{2}\right)-\xi^{sv}\left(G^{⋄}-e_{1}\right)=\frac{n+2}{n-2}-\frac{4(n-1)}{\left(n-\alpha )(n-\alpha -1\right)}. \end{array}$$

Let $f(x)=\frac{n+2}{n-2}-\frac{4(n-1)}{\left(n-x)(n-x-1\right)}(2\leq x\leq n-2)$ be a function. Taking derivation for *f*(*x*), we have

$$\begin{array}{c} \frac{df(x)}{dx}=-[\frac{4(n-1)}{{\left(n-x\right)}^{2}\left(n-x-1\right)}+\frac{4(n-1)}{\left(n-x\right){\left(n-x-1\right)}^{2}}]<0. \end{array}$$

So *f*(*x*) is a strict decreasing function. It is easy to verify that *f*(*x*) ≥ 0 if $x\leq \lfloor n-\frac{1+\sqrt{\frac{16n^{2}-47n+34}{n+2}}}{2}\rfloor$ and *f*(*x*) ≤ 0 if $x\geq \lceil n-\frac{1+\sqrt{\frac{16n^{2}-47n+34}{n+2}}}{2}\rceil$, which implies the result.

A *covering* of a graph *G* is a subset *K* of *V*(*G*) such that every edge of *G* has at least one end-vertex in *K*. The *covering number *γ** of a graph *G* is the number of vertices in any minimal covering. Since the complement of the maximum independent set is just the minimum covering, we have the following result.

**Theorem 5** *Let G be a connected graph on n vertices with covering number γ. Then*

$$\begin{array}{c} \xi^{sv}\left(G\right)\geq \gamma +\frac{2(n-\gamma )(2n-\gamma -2)}{\gamma }, \end{array}$$

*with equality holding if and only if* $G≅K_{\gamma }\vee \bar{K_{n-\gamma }}$.

A *vertex cut* of *G* is a subset *V*′ of *V* such that *G*−*V*′ is disconnected. A *κ*-vertex cut is a vertex cut of *κ* elements. The *vertex connectivity* of a graph *G*, denoted by *κ*(*G*), is the minimum *κ* for which *G* has a *κ*-vertex cut.

**Theorem 6** *Let G be a connected graph with n vertices and vertex connectivity κ. Then*

$$\begin{array}{c} \xi^{sv}\left(G\right)\geq 2n-\kappa +4\left(n-\kappa -1\right)\left(\frac{1}{\kappa }+\frac{1}{n-2}\right), \end{array}$$

*with equality holding if and only if G* ≅ *K*_*κ*_∨(*K*_1_∪*K*_*n*−*κ*−1_).

*Proof.* Let *G*_0_ be a graph with the minimal adjacent eccentric distance sum index among all connected graphs on *n* vertices with vertex connectivity *κ*. Then there exists a vertex cut *S* ⊂ *V*(*G*_0_) with |*S*| = *κ* such that *G*_0_−*S* = *G*_1_∪*G*_2_∪⋯∪*G*_*t*_, where *G*_1_, *G*_2_, ⋯, *G*_*t*_ (*t* ≥ 2) are connected components of *G*_0_−*S*. Since adding edges will decrease the adjacent eccentric distance sum index, the following three claims hold: (1) *t* = 2; (2) the subgraphs *G*_1_, *G*_2_ and the induced subgraph *G*_0_[*S*] are complete; (3) any vertex of *G*_1_∪*G*_2_ is adjacent to any vertex in *S*. Let |*G*_*i*_| = *n*_*i*_ for *i* = 1,2 and *n*_1_ ≥ *n*_2_. Then *G*_0_ ≅ *K*_*κ*_∨(*K*_*n*_1__∪*K*_*n*_2__) and *n*_1_+*n*_2_ = *n*−*κ*.

By the definition of adjacent eccentric distance sum index, we have

$$\begin{array}{c} \xi^{sv}\left(G_{0}\right)=\kappa +2\left(n-\kappa \right)+4n_{1}n_{2}\left(\frac{1}{n-n_{1}-1}+\frac{1}{n-n_{2}-1}\right). \end{array}$$

Let $G_{0}^{\prime }=K_{\kappa }\vee (K_{n_{1}+1}\cup K_{n_{2}-1})$. Then we consider the difference

$$\begin{array}{ccc} \xi^{sv}\left(G_{0}\right)-\xi^{sv}\left(G_{0}^{\prime }\right) & = & 4n_{1}n_{2}\left(\frac{1}{n-n_{1}-1}+\frac{1}{n-n_{2}-1}\right)\\ & & -4\left(n_{1}+1\right)\left(n_{2}-1\right)\left(\frac{1}{n-n_{1}-2}+\frac{1}{n-n_{2}}\right)\\ & = & 4\frac{\left(n_{1}-n_{2}+1\right)\left(n+\kappa -2\right)\left(\kappa -1\right)\left(n-1\right)}{\left(n-n_{1}-1\right)\left(n-n_{2}-2\right)\left(n-n_{2}\right)\left(n-n_{2}-1\right)}>0. \end{array}$$

Therefore, *G* ≅ *K*_*κ*_∨(*K*_1_∪*K*_*n*−*κ*−1_) has the minimal adjacent eccentric distance sum index among all connected graphs on *n* vertices with vertex connectivity *κ*.

**Theorem 7** *Let G be a connected graph with n vertices and minimum degree δ. Then*

$$\begin{array}{c} \xi^{sv}\left(G\right)\geq 2n-\delta +4\left(n-\delta -1\right)(\frac{1}{\delta }+\frac{1}{n-2}), \end{array}$$

*with equality holding if and only if G* ≅ *K*_*δ*_∨(*K*_1_∪*K*_*n*−*δ*−1_).

*Proof.* Let *G* be a graph with the minimum adjacent eccentric distance sum index among connected graphs of order *n* with minimum degree *δ*. Assume that *G* has vertex connectivity *κ*.

Let $f(x)=2n-x+4(n-x-1)(\frac{1}{x}+\frac{1}{n-2})$ be a function. It can be checked that $\frac{df(x)}{dx}<0$ and therefore *f*(*x*) is strictly decreasing. Hence *f*(*κ*) ≥ *f*(*δ*) due to the fact that *κ* ≤ *δ*.

By Theorem 6, we have *ξ* ^*sv*^(*G*) ≥ *f*(*κ*) ≥ *f*(*δ*). If *ξ* ^*sv*^(*G*) = *f*(*δ*), then *κ* = *δ*. This implies the result.

Similarly, we have

**Theorem 8** *Let G be a connected graph on n vertices with edge connectivity λ. Then*

$$\begin{array}{c} \xi^{sv}\left(G\right)\geq 2n-\lambda +4\left(n-\lambda -1\right)(\frac{1}{\lambda }+\frac{1}{n-2}), \end{array}$$

*with equality holding if and only if G* ≅ *K*_*λ*_∨(*K*_1_∪*K*_*n*−*λ*−1_).

**Theorem 9** *Let G be a connected graph on n vertices with maximum degree* △. *Then*

$$\begin{array}{c} \xi^{sv}\left(G\right)\geq 2n-△+4\left(n-△-1\right)(\frac{1}{△}+\frac{1}{n-2}), \end{array}$$

*with equality holding if and only if G* ≅ *K*_△_∨(*K*_1_∪*K*_*n*−△−1_).

The *chromatic number* of a graph *G*, denoted by *χ*(*G*), is the minimum number of colors such that *G* can be colored with these colors and no two adjacent vertices have the same color.

**Theorem 10** *Let G a connected graph on n vertices with chromatic number χ. Assume that n* = *χs* + *r* *with* 1 ≤ *r* < *χ*. *Then*

$$\begin{array}{c} \xi^{sv}\left(G\right)\geq \frac{2s(\chi -r)(n+s-2)}{n-s}+\frac{2r(s+1)(n+s-1)}{n-s-1}, \end{array}$$

*with equality holding if and only if G* ≅ *T*_*n*,*χ*_.

*Proof.* Let *G*_0_ be a *χ*-chromatic graph with minimal adjacent eccentric distance sum index. *G*_0_ must be of the form $\bar{K_{1}}\vee \cdots \vee \bar{K_{\chi }}$. For any vertex $v\in V(\bar{K_{i}})$, *ɛ*(*v*) = 2, *D*(*v*) = *n*+*n*_*i*_−2 and *deg*(*v*) = *n*−*n*_*i*_. So we have

$$\begin{array}{c} \xi^{sv}\left(G_{0}\right)=\sum_{i=1}^{\chi }\frac{2n_{i}\left(n+n_{i}-2\right)}{n-n_{i}}. \end{array}$$

Assume that *n*_1_ ≤ *n*_2_⋯ ≤ *n*_*χ*_. Let $G_{0}^{\prime }=\bar{K_{1}}\vee \cdots \vee \bar{K_{n_{i}+1}}\vee \cdots \vee \bar{K_{n_{j}-1}}\vee \cdots \vee \bar{K_{\chi }}$ if 2 ≤ *n*_*i*_ < *n*_*j*_.

Considering the difference

$$\begin{array}{ccc} \xi^{sv}\left(G_{0}\right)-\xi^{sv}\left(G_{0}^{\prime }\right) & = & \frac{2n_{i}\left(n+n_{i}-2\right)}{n-n_{i}}+\frac{2n_{j}\left(n+n_{j}-2\right)}{n-n_{j}}\\ & & -\frac{2\left(n_{i}+1\right)\left(n+n_{i}-1\right)}{n-n_{i}-1}-\frac{2\left(n_{j}-1\right)\left(n+n_{j}-3\right)}{n-n_{j}+1}\\ & = & -\frac{4n\left(n-1\right)\left(n_{j}-n_{i}-1\right)\left(n_{i}+n_{j}-2n\right)}{\left(n-n_{i}\right)\left(n-n_{i}-1\right)\left(n-n_{j}\right)\left(n-n_{j}+1\right)}\geq 0. \end{array}$$

Therefore, *T*_*n*,*χ*_ has the minimal adjacent eccentric distance sum index among all connected graphs on *n* vertices with chromatic number *χ*. By simple calculations, we have $\xi^{sv}(T_{n,\chi })=\frac{2s(\chi -r)(n+s-2)}{n-s}+\frac{2r(s+1)(n+s-1)}{n-s-1}.$

A *diametrical path* in a graph is a path whose length equals the diameter of the graph. Let *P*_*D*+1_ = *v*_0_ *v*_1_…*v*_*D*_ be a diametrical path in *G*. For the vertex *v*_0_, the distance layer *L*_*i*_ is the set of vertices *v* ∈ *G* satisfying *d*(*v*_0_, *v*) = *i* for *i* = 0, 1, …, *D*, i. e.

$$\begin{array}{c} L_{i}=\left\{v\;|\;d\left(v_{0},v\right)=i\right\},\;i=0,1,\ldots ,D. \end{array}$$

In particular, $L_{\left\lceil \frac{D}{2}\right\rceil }$ is called *the central layer*.

The distance layer *L*_*i*_ is called *trivial* if |*L*_*i*_| = 1. Obviously, *L*_0_ is a trivial layer.

For $\bar{K_{2}}=\{u,v\}$, |*l*_1_−*l*_2_| ≤ 1 and *l*_1_+*l*_2_ = *D*−2, the graph $G_{D}^{*}$ is obtained from $K_{n-D}⋁\bar{K_{2}}$ (the join of *K*_*n*−*D*_ and $\bar{K_{2}}$) by identifying one end-vertex of each path of length *l*_1_ and *l*_2_ with *u* and *v*, respectively. It is evident that its noncentral layers are trivial and any subgraph induced by the union of the central layer and its neighboring layer is complete.

For odd *D*, denote by ${𝓖}_{D}^{⋄}$ the set of graphs obtained from *K*_*k*_⋁*K*_*n*−*D*−1−*k*_ by connecting an end-vertex of a path *P*_1_ = *P*_(*D*−1)/2_ with all vertices from *K*_*k*_ and connecting an end-vertex of a path *P*_2_ = *P*_(*D*−1)/2_ with all vertices from *K*_*n*−*D*−1−*k*_.

**Theorem 11** *Let G be a connected graph of order n with diameter D. Then*

$$\begin{array}{c} \xi^{sv}\left(G\right)\geq \xi^{sv}\left(G_{D}^{*}\right) \end{array}$$

*with equality holding if and only if* $G≅G_{D}^{*}$ *for even* *D* *and* $G\in {𝓖}_{D}^{⋄}$ *for odd D*.

*Proof.* Let *G*_0_ be a graph with the minimal adjacent eccentric distance sum index among all connected graphs on *n* vertices with diameter *D*. Assume that *P*_*D*+1_ = *v*_0_ *v*_1_⋯*v*_*D*_ is a diametrical path in *G*_0_. Since adding an edge decreases the adjacent eccentric distance sum index, the subgraphs induced by the union of two neighboring distance layers in *G*_0_ are complete.

Suppose that $k\in \{1,\cdots ,\lceil \frac{D+1}{2}\rceil -1\}$ is the smallest number such that |*V*_*k*_| > 1 in *G*_0_. Let *v* be a vertex in *V*_*k*_, but different to *v*_*k*_. We construct a new graph $G_{0}^{\prime }$ from *G*_0_ by removing *v* from *V*_*k*_ to *V*_*k*+1_ such that $G_{0}^{\prime }$ has the diameter *D* and the subgraphs induced by the union of two neighboring distance layers in $G_{0}^{\prime }$ are complete. By the definition of adjacent eccentric distance sum index, we have

$$\begin{array}{ccc} \xi^{sv}\left(G_{0}\right) & = & \sum_{u\in V_{0}\cup \cdots \cup V_{k-1}}\frac{\varepsilon (u)D(u)}{deg(u)}+\sum_{u\in V_{k}-\left\{v\right\}}\frac{\varepsilon (u)D(u)}{deg(u)}\\ & & +\sum_{u\in V_{k+1}\cup \cdots \cup V_{D}}\frac{\varepsilon (u)D(u)}{deg(u)}+\frac{\varepsilon (v)D(v)}{deg(v)};\\ \xi^{sv}\left(G_{0}^{\prime }\right) & = & \sum_{u\in V_{0}\cup \cdots \cup V_{k-1}}\frac{\varepsilon (u)(D(u)+1)}{deg(u)}+\sum_{u\in V_{k}-\left\{v\right\}}\frac{\varepsilon (u)D(u)}{deg(u)}\\ & & +\sum_{u\in V_{k+1}\cup \cdots \cup V_{D}}\frac{\varepsilon (u)(D(u)-1)}{deg(u)}+\frac{\left(\varepsilon \left(v\right)-1\right)(D\left(v\right)-n+|V_{k}|+|V_{k+1}|+2k)}{deg(v)}. \end{array}$$

It follows that

$$\begin{array}{ccc} \xi^{sv}\left(G_{0}\right)-\xi^{sv}\left(G_{0}^{\prime }\right) & > & -\sum_{u\in V_{0}\cup \cdots \cup V_{k-1}}\frac{\varepsilon (u)}{deg(u)}+\sum_{u\in V_{k+1}\cup \cdots \cup V_{D}}\frac{\varepsilon (u)}{deg(u)}\\ & = & (\sum_{u\in V_{D}}\frac{\varepsilon (u)}{deg(u)}-\sum_{u\in V_{0}}\frac{\varepsilon (u)}{deg(u)})+\cdots \\ & & +(\sum_{u\in V_{D-k+1}}\frac{\varepsilon (u)}{deg(u)}-\sum_{u\in V_{k-1}}\frac{\varepsilon (u)}{deg(u)})\\ & & +\sum_{u\in V_{k+1}\cup \cdots \cup V_{D-k}}\frac{\varepsilon (u)}{deg(u)}. \end{array}$$

Note that for any 1 ≤ *i* ≤ *k*−1, we have *ɛ*(*u*) = *ɛ*(*w*) for *u* ∈ *V*_*i*_, *w* ∈ *V*_*D*−*i*_ and

$$\begin{array}{ccc} \sum_{w\in V_{D-i}}\frac{\varepsilon (w)}{deg(w)}-\sum_{u\in V_{i}}\frac{\varepsilon (u)}{deg(u)} & = & \left(deg\left(w\right)-1\right)\frac{\varepsilon (w)}{deg(w)}-\frac{\varepsilon (u)}{2}\\ & = & \left(\frac{deg(w)-1}{deg(w)}-\frac{1}{2}\right)\varepsilon \left(w\right)\geq 0. \end{array}$$

For *i* = 0, we have

$$\begin{array}{c} \sum_{w\in V_{D}}\frac{\varepsilon (w)}{deg(w)}-\sum_{u\in V_{0}}\frac{\varepsilon (u)}{deg(u)}=\varepsilon \left(w\right)-\varepsilon \left(u\right)=0. \end{array}$$

It follows that

$$\begin{array}{ccc} \xi^{sv}\left(G_{0}\right)-\xi^{sv}\left(G_{0}^{\prime }\right) & > & \sum_{u\in V_{k+1}\cup \cdots \cup V_{D-k}}\frac{\varepsilon (u)}{deg(u)}>0. \end{array}$$

This contradict to the fact that *G*_0_ has the minimal adjacent eccentric distance sum index in 𝓖_*n*,*D*_.

Suppose that $k\in \{\lceil \frac{D+1}{2}\rceil +1,\cdots ,D-1\}$ is the largest number such that |*V*_*k*_| > 1. Let *w* be a vertex in *V*_*k*_, but different to *v*_*k*_. We construct a new graph $G_{0}^{\prime \prime }$ from *G*_0_ by removing *w* from *V*_*k*_ to *V*_*k*−1_ such that $G_{0}^{\prime \prime }$ has the diameter *D* and the subgraphs induced by the union of two neighboring distance layers in $G_{0}^{\prime \prime }$ are complete. As above, we can get that $\xi^{sv}(G_{0})>\xi^{sv}(G_{0}^{\prime \prime })$, a contradiction.

Therefore, $G_{D}^{*}$ is the unique graph with minimal adjacent eccentric distance sum index in 𝓖_*n*,*D*_ for even *D*; for odd *D*, it can be seen that all graphs in ${𝓖}_{D}^{⋄}$ have the same value $\xi^{sv}(G_{D}^{*})$ and there are exactly $\left\lceil \frac{n-D}{2}\right\rceil$ extremal graphs with minimal adjacent eccentric distance sum index.

At last we shall calculate the value $\xi^{sv}(G_{D}^{*})$. Assume that *P*_*D*+1_ = *v*_0_ *v*_1_⋯*v*_*D*_ be a diametrical path in $G_{D}^{*}$. We divide the vertex set $V(G_{D}^{*})$ into three parts: $V_{1}=\{v_{0},v_{1},\cdots ,v_{\lceil \frac{D}{2}\rceil -1}\}$, $V_{2}=\{v_{\lceil \frac{D}{2}\rceil +1},\cdots ,v_{D}\}$ and the central layer, i.e., $V_{3}=V_{\left\lceil \frac{D}{2}\right\rceil }$.

When *D* is even, we have

- For *v*_*i*_ ∈ *V*_1_(*i* ≥ 1), we have: *ɛ*(*v*_*i*_) = *D*−*i*,
$D(v_{i})=\frac{i(i+1)}{2}+\frac{\left(D-i)(D-i+1\right)}{2}+(\frac{D}{2}-i)(n-D-1)$, *deg*(*v*_*i*_) = 2; *ɛ*(*v*_0_) = *D*,
$D(v_{0})=\frac{\left(D)(D+1\right)}{2}+(\frac{D}{2})(n-D-1)$, *deg*(*v*_0_) = 1
- For *v*_*i*_ ∈ *V*_2_, we have: *ɛ*(*v*_*i*_) = *i*,
$D(v_{i})=\frac{i(i+1)}{2}+\frac{\left(D-i)(D-i+1\right)}{2}+(i-\frac{D}{2})(n-D-1)$, *deg*(*v*_*i*_) = 2; *ɛ*(*v*_*D*_) = *D*,
$D(v_{D})=\frac{D(D+1)}{2}+(D-\frac{D}{2})(n-D-1)$, *deg*(*v*_*D*_) = 1
- For *v* ∈ *V*_3_, we have: $ɛ(v)=\frac{D}{2}$, $D(v)=\frac{D(D+2)}{4}+n-D-1$, *deg*(*v*) = *n*−*D*+1.

So we have

$$\begin{array}{ccc} \xi^{d}\left(G_{D}^{*}\right) & = & \frac{1}{2}\sum_{i=1}^{\frac{D}{2}-1}\left(D-i\right)[\frac{i(i+1)}{2}+\frac{\left(D-i)(D-i+1\right)}{2}+\left(\frac{D}{2}-i\right)\left(n-D\right)]\\ & & +D[\frac{\left(D)(D+1\right)}{2}+\left(\frac{D}{2}\right)\left(n-D-1\right)]\\ & & +\frac{1}{2}\sum_{j=\frac{D}{2}+1}^{D-1}j[\frac{j(j+1)}{2}+\frac{\left(D-j)(D-j+1\right)}{2}+\left(j-\frac{D}{2}\right)\left(n-D\right)]\\ & & +D[\frac{D(D+1)}{2}+\left(D-\frac{D}{2}\right)\left(n-D-1\right)]\\ & & +\frac{1}{n-D+1}\frac{D(n-D)}{2}[\frac{D(D+2)}{4}+n-D-1]\\ & = & -\frac{D(5D^{4}+\left(15n+43\right)D-\left(20n^{2}-76n+140\right)D^{2})}{192(n-D+1)}\\ & & +\frac{D(\left(144n^{2}-84n+28\right)D+112n^{2}-80n)}{192(n-D+1)}. \end{array}$$

Similarly, if *D* is odd, for any $G\in {𝓖}_{D}^{⋄}$, one has

$$\begin{array}{ccc} \xi^{d}\left(G\right) & = & \frac{1}{2}\sum_{i=1}^{\frac{D-1}{2}}\left(D-i\right)[\frac{i(i+1)}{2}+\frac{\left(D-i)(D-i+1\right)}{2}+\left(\frac{D+1}{2}-i\right)\left(n-D-1\right)]\\ & & +D[\frac{\left(D)(D+1\right)}{2}+\left(\frac{D+1}{2}\right)\left(n-D-1\right)]\\ & & +\frac{1}{2}\sum_{i=\frac{D+3}{2}}^{D-1}i[\frac{i(i+1)}{2}+\frac{\left(D-i)(D-i+1\right)}{2}\\ & & +i\left(n-D-1\right)-\frac{D+1}{2}\left(n-D-1\right)]\\ & & +D[\frac{D(D+1)}{2}+\left(D-\frac{D+1}{2}\right)\left(n-D-1\right)]\\ & & +\frac{1}{n-D+1}\left(n-D\right)\frac{D+1}{2}[\frac{\left(D+1)(D+3\right)}{8}+\frac{\left(D-1)(D+1\right)}{8}+n-D-1]\\ & = & -\frac{5D^{5}+\left(15n+23\right)D^{4}-\left(20n^{2}+96n-66\right)D^{3}-\left(144n^{2}-42n+134\right)D^{2}}{192(n-D+1)}\\ & & +\frac{\left(124n^{2}-240n+35\right)D+96n^{2}-87n-15}{192(n-D+1)}. \end{array}$$

## Discussion

In this paper, we investigated the adjacent eccentric distance sum index and gave some lower bounds for this index in terms of some graph parameters such as independence number, covering number, vertex connectivity, chromatic number, diameter and some other topological indices. Moreover, the extremal graphs (attaining these bounds) are also characterized. Throughout this paper, the proof techniques used the structural properties of the graph parameters and the topological indices.

We only considered several graph parameters and topological indices in this work. To complete this knowledge, we shall consider relations between the adjacent eccentric distance sum index and other graph parameters and topological indices. In the future, we will continue to study extremal values of adjacent eccentric distance sum index for some special classes of graphs as well as for more general graphs. One direction of the future work is to consider the extremal values among some chemical or biology networks, such as benzenoid systems, fullerene graphs and polymeric networks.
